# Diagnosing eyewitness identifications with reaction time-based concealed information test: the effect of observation time

**DOI:** 10.1007/s00426-022-01643-5

**Published:** 2022-02-08

**Authors:** Melanie Sauerland, Dave Koller, Astrid Bastiaens, Bruno Verschuere

**Affiliations:** 1 Department of Clinical Psychological Science, Section Forensic Psychology, Faculty of Psychology and Neuroscience, Maastricht University, P.O. Box 616, Maastricht, 6200 MD The Netherlands; 2 Department of Clinical Psychology, University of Amsterdam, Amsterdam, The Netherlands; 3 Department of Psychology, University of Zurich, Zurich, Switzerland

## Abstract

Eyewitness identification procedures rely heavily on explicit identification from lineups. Lineups have been criticized because they have a considerable error rate. We tested the potential of implicit identifications in a Concealed Information Test (CIT) as an alternative. Previous experiments have suggested that implicit identification procedures might be suited when viewing conditions were favorable. In two experiments, mock eyewitnesses (*N*s = 94, 509) witnessed a videotaped mock theft with longer or shorter observation time. We derived eyewitness identifications either implicitly from CIT reaction times or explicitly from simultaneous photo lineups. In Experiment 2, we also manipulated perpetrator presence. In both experiments, the perpetrator-present CIT showed capacity to diagnose face recognition, with large effect sizes (*d*_E1_ = 0.85 [0.51; 1.18]; *d*_E2_ = 0.74 [0.52; 0.96]), as expected. Unexpectedly, no moderation by observation time was found. In line with our hypothesis, no CIT effect emerged in the perpetrator-absent condition, indicating the absence of recognition (*d*_E2_ = 0.02 [− 0.17; 0.20]). We found no compelling evidence that one method would outperform the other. This work adds to accumulating evidence that suggests that, under favorable viewing conditions and replication provided, the RT-CIT might be diagnostic of facial recognition, for example when witnesses are hesitant of making an explicit identification. Future work might investigate conditions that affect performance in one, but not the other identification method.

**Supplementary Information:**

The online version contains supplementary material available at 10.1007/s00426-022-01643-5.

## Introduction

Eyewitnesses often play an important role in the investigation of crime. They testify to the course of events and the identity of perpetrators. Current procedures for establishing the identity of a perpetrator largely rely on explicit identification from lineups or showups. Fifty years of laboratory, field, and archival research have shown error rates for lineups and showups are as high as 50% on average (e.g., Clark et al., 2008; Fitzgerald & Price, 2015) and case studies have painfully demonstrated that misidentifications can lead to wrongful convictions. Examples of such wrongful convictions are known all across North America and Europe (Christianson et al., 1992; Davies & Griffiths, 2008; Garrett, 2011; Lindemans, 2019; Thompson-Cannino et al., 2009; van Koppen & van der Horst, 2006). As a result, some countries dismiss explicit identification procedures altogether (e.g., South Korea, Indonesia) and scholars have started to question researchers’ sustained commitment to traditional lineups (Brewer & Wells, 2011; Wells et al., 2006) and called for the development of radically different procedures (Dupuis & Lindsay, 2007). Indirect measures—being less intentional, faster, and more stimulus driven—might probe for such a radical alternative. The Concealed Information Test (CIT; Lykken, 1959) provides such an indirect assessment of recognition. We conducted two experiments that tested the usefulness of the CIT as a means of diagnosing face recognition when encoding conditions are favorable.

The CIT is a well-established technique for memory detection (Lykken, 1959; for a review see Verschuere et al., 2011). Similar to a lineup, a CIT consists of several stimuli, only one of which is crime related (e.g., stolen goods: 500 EUR banknote), embedded in a series of plausible, yet crime-unrelated answers (e.g., a cell phone, a credit card, a laptop, a Rolex). However, rather than relying on explicit responses, the CIT infers recognition from indirect measures, such as skin conductance, respiration, and the P300 event-related potential. In our above example, police could ask the suspect about the stolen goods: Was it … A cell phone? … A credit card? …A laptop? … A Rolex? … A 500 EUR banknote? Stronger physiological reactions to the actual stolen banknote, compared to other items, indicate concealed recognition. When combining multiple questions, for example about a weapon, the crime scene, and the location of the crime, the CIT can detect recognition with high validity. The diagnostic efficiency of the CIT, measured as the area under the receiver operating characteristic curve, is about 0.82–0.94 (Meijer et al., 2016). In other words, a randomly chosen person who experiences recognition for the critical stimulus has an 82–94% chance to respond stronger to it in the CIT than a randomly chosen person who does not experience recognition. In addition, a meta-analysis reported large effect sizes for different physiological measures, varying between *d* = 0.89 and 1.89 (Meijer et al., 2014).

Recently, reaction times have enjoyed increasing popularity as the response measure in the CIT (for a theoretical analysis, see Verschuere & De Houwer, 2011; for a meta-analytic review, see Suchotzki et al., 2017). Reaction times constitute an attractive outcome measure because their use is resource friendly, requiring only a single computer, and Web-based testing is possible with high reliability and validity (Kleinberg & Verschuere, 2015). In the reaction time-based CIT (RT-CIT), the stimuli appear sequentially on the computer screen for a brief interval. The three different types of stimuli are probes, irrelevants, and targets. The *probe* is the crime-related stimulus, and the *irrelevants* are foils. Participants learn that they should press one of two keys when they see the probe or an irrelevant as fast and as accurately as possible. The other key is reserved for the so-called *targets*.^1^ Targets are non-crime-related stimuli that participants study just before the test. The use of a response deadline prevents strategic slowing (Suchotzki et al., 2021). Building on the example above, participants may be instructed that the CIT will examine recognition of stolen goods and asked to press the YES button whenever encountering the target (e.g., a laptop) and the NO button for all other items. For innocent (uninformed) participants, all NO-reaction times should be similar. For guilty (informed) participants, the option *500 EUR banknote* should stand out and affect participants’ response. Longer reaction times for *500 EUR banknote* than the other NO responses provide an index of recognition. A recent meta-analysis reported a large effect size of Cohen’s *d* = 1.04 (corrected), confirming the diagnostic efficiency of the RT-CIT (Suchotzki et al., 2017).

The association between recognition and reaction times is theorized to result from familiarity-based responding. After all, the most efficient way to take an RT-CIT is to rely on familiarity—a fast and automatic process (Yonelinas, 2002). For innocent (uninformed) participants, familiarity-based responding leads to correct responses for all stimuli (target: YES, probe: NO, irrelevant: NO). For guilty (informed) participants, familiarity-based responding leads to the correct response for targets and irrelevants, whereas for probes, familiarity (YES, recognized) conflicts with the responses required by the task (NO, not the target). It is this conflict and the required control to override it that slows down the response. Increases in the RT-CIT effect for interventions promoting familiarity-based responding, such as using familiar targets or adding familiarity-related fillers, support this idea (Lukács et al., 2017; Suchotzki et al., 2018). Furthermore, probe processing is associated with measures indexing response conflict and resolution (e.g., Hadar et al., 2012, 2019; Seymour & Schumacher, 2009; Suchotzki et al., 2015).

First support for the idea that indirect measures in general and the CIT in particular can provide information about *face* recognition came from two studies with pre-school and school children (Newcombe & Fox, 1994; Stormark, 2004). Participants viewed slides of playmates or former classmates and unfamiliar faces, with their skin conductance, heart rate, or both being recorded. Participants also provided direct face recognition responses. Direct and indirect measures scored above chance in both studies, but the indirect measures outperformed direct recognition decisions. In the first application of the CIT for the purpose of identifying incidentally encountered faces, participants watched four mock crimes across two testing sessions (Lefebvre et al., 2007). In the perpetrator-present conditions, participants sequentially viewed the photograph of the perpetrator, the victim, and five foils, while EEG recordings were made. Deviating from the classic CIT procedure, participants had three response options, indicating that a picture depicted the perpetrator, the victim, or another person. In other words, participants made an explicit identification in an ERP-based CIT. The CIT revealed recognition of the perpetrator, as did explicit identification. While the results point to the potential of the CIT for cooperative eyewitness identification, the electrophysiological index of recognition may have been evoked by the explicit identification. Additionally, Lefebvre et al.’s facial stimuli (2007, 2009) were matched for gender, age, race, and hair length, but it is unclear in how far the stimuli matched in terms of hair color or hair style, and no measures of effective lineup size were provided.

Recent investigations applied a stricter CIT protocol in a typical eyewitness paradigm to assess the usefulness of the CIT as an alternative to classic eyewitness identification procedures (Georgiadou et al., 2019; Sauerland et al., 2019). Participants viewed a filmed mock crime and then took an RT-CIT. To ensure the fairness of the CIT, the included pictures were selected with the same procedure that is considered best practice for selecting lineup fillers (Doob & Kirshenbaum, 1973; cf. Wells et al., 1998, 2020). In one experiment, the CIT showed a good capacity to differentiate the film actors (i.e., probes) from fillers (*d* = 1.21; Georgiadou et al., 2019, Experiment 2b) and moderate capacity in another (*d* = 0.39; Sauerland et al., 2019, Experiment 4). Yet, the average effect across five experiments revealed a negligibly small overall effect size of *d* = 0.14 (Sauerland et al., 2019).

The RT-CIT effect sizes in the eyewitness identification field tend to be smaller than typically reported for RT-CIT experiments (i.e., *d* = 1.04 in the meta-analysis by Suchotzki et al., 2017). One reason for this finding is that the probes in a facial identification RT-CIT protocol have to match the irrelevants more closely than in other fields (Georgiadou et al., 2019, Experiment 1; Sauerland et al., 2019, Experiment 5). This is necessary for an identification procedure to be fair (Wells et al., 2020). Differences in encoding conditions and event complexity offer an explanation for the conflicting findings *within* facial identification RT-CIT experiments. More specifically, the experiments with moderate to large effects included only two rather than four actors and provided ample close-ups of both (Georgiadou et al., 2019, Experiment 2b; Sauerland et al., 2019, Experiment 4). In Georgiadou et al., (2019, Experiment 2b) encoding was additionally enhanced by presenting the pictures of the actors for 15 s after participants had viewed the stimulus film and prior to taking the RT-CIT. From an applied eyewitness recognition perspective, this setup was somewhat flawed, though, because the presented picture was identical to the picture used in the CIT (Burton, 2013). Nevertheless, the two experiments combined suggest that observation time might be key for applying the RT-CIT in face recognition. Observation time is associated with initial memory strength and predictive of face recognition performance (Bornstein et al., 2012). It is possible that a certain degree of memory strength is required to ensure reliable performance in the CIT. Although observation time is not under the control of investigators, this finding might be useful in cases with known long observation time.

In two preregistered experiments, we manipulated overall observation time, close-up duration, and facial viewing time during encoding to test whether encoding conditions critically determine the validity of the RT-CIT as an index of face recognition. Extending prior work, we included a classic lineup condition as a benchmark of eyewitness performance. In Experiment 1, participants viewed a stimulus film with shorter or longer observation time before completing an RT-CIT or making lineup decisions from probe-present lineups. In Experiment 2, we increased the observation time difference across conditions further and added probe-absent conditions to test in how far insights from previous work (Georgiadou et al., 2019; Sauerland et al., 2019) apply to a situation where the suspect is in fact innocent. We expected a stronger CIT effect (i.e., difference in reaction times to probes vs. irrelevants) when observation time was longer, rather than shorter (CIT encoding effect; hypothesis 1). In Experiment 2, we additionally hypothesized a larger CIT effect in the probe-present compared to the probe-absent condition (hypothesis 2). In Experiment 2, we also predicted that identification performance with lineups would vary as a function of observation time (lineup encoding effect; hypothesis 3). The relative capacity of the CIT and lineups to diagnose face recognition is of strong applied interest, but we had no hypotheses for this comparison.

## Method

Both experiments received ethical approval by the Ethics Review Committee of the faculty (approval codes 160_03_01_2016_S3 and 231_140_12_2020_S1) and were preregistered on the open science framework (Experiment 1: https://osf.io/xcdq4; Experiment 2: https://osf.io/sb38g). The inquisit scripts and data can also be found on these publicly available links: https://osf.io/u6gnt/; https://osf.io/b9ucn/. We cannot share the videos and pictures separately because we do not have permission of the individuals involved.

### Participants

#### Power analyses

To establish the required sample size for the CIT conditions in Experiment 1, we conducted a G*Power analysis (Faul et al., 2007, 2009) for repeated measures ANOVA with a within–between interaction. We based the estimate for the expected effect size on Georgiadou et al., (2019; Experiment 1, matched condition; *d* = 0.50, i.e., *f* = .25) and set *α* to .05 and power to .95.^2^ The number of groups and measurements used for the analysis were each two. Correlation among repeated measures was taken from Sauerland et al., (2019: *r* = .7). This analysis resulted in a sample size of only 34 participants for the CIT conditions (17 per group). Due to this low sample size, we decided to use a somewhat larger sample size of 46 participants, with 23 participants in the two CIT between-subjects conditions. Because our comparison between the lineup and CIT conditions was exploratory and because we had no hypothesis about the size or direction of the effect, we simply matched the cell sizes of the two lineup conditions with the CIT conditions (i.e., total *N* = 23 × 4 = 92).

In Experiment 2, we wanted to improve statistical power, by increasing both the strength of the observation time manipulation and the sample size. We based our power analysis on the comparison between CIT and lineup performance because the power of this comparison is weakest. We entered *df* = 1, *α* = .05, power = .95, and *φ* = .30 for a chi-square test, to be able to pick up at least moderate differences between lineup and CIT. With a power of .95, the required sample size for each of the four comparisons (probe presence: absent vs. present by observation time: shorter vs. longer) was 145 per comparison and 580 in total. Accounting for 10% dropout or exclusions, we decided to test 638 participants in total. Due to simultaneous starts in the online portal, the actual number of participants who started the study was slightly larger, as mentioned in the preregistration. Although attrition was larger than anticipated (final *N* = 509, see below for details), power for the comparisons between CIT and lineups was still high, ranging from 0.90 to 0.94 (*n*s between 116 and 135 per comparison), assuming a moderate effect size.

#### Samples

In Experiment 1, we excluded 3 of 97 participants because they did not pass the attention check or because they gave wrong responses to all target trials in the CIT condition. The final sample consisted of *N* = 94 participants. Table 1 shows the distribution of the sample across the four conditions. Participants (*M*_age_ = 24.41 years, *SD* = 7.21, range 18–57 years) were mostly women (77.7%) and bachelor’s (59.1%) or master’s students (15.1%) who studied at the Faculties of Psychology and Neuroscience (68.1%), Arts and Sciences, Science and Engineering (5.8% each), Law (4.3%), or other (15.9%).

**Table 1:** Correct classification rate of indirect identifications with the RT-CIT and identification accuracy in traditional lineups as a function of observation time in two experiments

Experiment 1 (*N* = 94)	Standard observation time		CIT vs. lineup (Bayes factor)		Enhanced observation time		CIT vs. lineup (Bayes factor)	
	CIT (*n* = 23) correct classification rate (%)	Lineup (*n* = 22) identification accuracy (%)			CIT (*n* = 24) correct classification rate (%)	Lineup (*n* = 25) identification accuracy (%)		
			BF_10_	BF_01_			BF_10_	BF_01_
Thief probe- present	30.4	63.6	4.01		54.2	68.0		1.85
Victim probe- present	39.1	36.4		2.86	58.3	40.0		1.32

Experiment 2 (*N* = 509)	Shorter observation time		CIT vs. lineup (Bayes factor)		Longer observation time		CIT vs. lineup (Bayes factor)	
	CIT (*n* = 109) correct classification rate (%)	Lineup (*n* = 148) identification accuracy (%)			CIT (*n* = 105) correct classification rate (%)	Lineup (*n* = 147) identification accuracy (%)		
			BF_10_	BF_01_			BF_10_	BF_01_
Thief probe-present	54.2	36.5	1.40		58.5	49.3		2.71
Victim probe-present	43.8	42.7		4.48	45.3	52.3		3.31
Thief probe-absent	65.5	63.5		4.76	71.2	86.5	1.61	
Victim probe-absent	70.5	53.0	1.60		75.0	75.6		5.28

The Bayes factor BF_10_ expresses how much more likely the data are under the hypothesis of a difference in accuracy of the CIT vs. the lineup as compared to the null hypothesis of no difference in accuracy. If the evidence supports the null hypothesis (i.e., BF_10_ < 1), we present BF_01_ for ease of interpretation. BF_01_ expresses how much more likely the data are under the null hypothesis as compared to the alternative hypothesis of a difference in accuracy of the CIT vs. the lineup

In Experiment 2, there were 704 participants. Of these, 127 terminated participation prematurely, 42 failed the attention check, and 26 exceeded the acceptable error rate (> 50%) for one of the three CIT trial types (probes, irrelevants, targets). The final sample hence consisted of *N* = 509 participants, 295 in the lineup conditions and 214 in the CIT conditions. The smaller sample size in the CIT compared to the lineup conditions can probably be attributed to the fact that participants had to download a plug-in for the Web-based software used to run the RT-CIT. Despite explanation in the pre-study information, participants might have been wary of the download. Table 2 shows the distribution of the sample across the eight conditions. Participants (*M*_age_ = 26.29 years, *SD* = 8.41, range 18–59 years; *n* = 1 missing) were mostly men (59.8%) and self-identified as White/Caucasian (99.6%; Latino/Hispanic: 0.2%; *n* = 1 missing). Participants were students (58.3%; *n* = 5 missing), full-time employed (28.0%), unemployed and job seeking (27.6%), or 'other' (22.4%; *n* = 10 missing). The most common nationalities were Portuguese (27.0%), Polish (22.8%), Italian (13.0%), British (12.8%), and Greek (7.3%; *n* = 3 missing).

**Table 2:** Distribution of the sample (*n*) across eight conditions as a function of identification procedure, probe presence, and observation time in Experiment 2 (*N* = 509)

	Lineup (*n* = 295)			RT-CIT (*n* = 214)		
	Observation time		Across observation time conditions	Observation time		Across observation time conditions
	Shorter	Longer		Shorter	Longer	
Thief probe-present	74	73	147	48	53	101
Victim probe-present	82	65	147			
Thief probe-absent	74	74	148	61	52	113
Victim probe-absent	66	82	148			

### Design

In Experiment 1, we used a 2 (observation time: standard vs. enhanced) × 2 (identification procedure: CIT vs. lineup) between-subjects design. Experiment 2 had a 2 (observation time: shorter vs. longer) × 2 (identification procedure: CIT vs. lineup) × 2 (probe presence: absent vs. present) between-subjects design.

The dependent measures in the CIT were the reaction times to probes and irrelevants in each CIT condition. Identification decisions were coded as accurate (hits, correct rejections) or inaccurate (foil selection, false rejections, misses).

### Materials

#### Stimulus films

We used variations of Sauerland et al.’s (2019, Experiment 4) stimulus films to create two observation time conditions in both experiments. All eight film versions (four per experiment) depicted the theft of a cell phone, involving a thief and a victim. The roles of the two female actors (thief or victim) were counterbalanced across observation time conditions. The films were without audio.

Table 3 shows an overview of observation time and facial viewing time per actor across film versions. In Experiment 1, overall observation time in the standard observation time condition was 29–36 s for each actor. Frontal face views lasted for 9–12 s for thieves and 7–9 s for victims. For the enhanced observation time condition, we added previously unused coverage of the events, slowed down some of the coverage, and included stills of 15 s of each actor. In these enhanced films, the overall observation time was 51–54 s for thieves and 45 s for victims and frontal face views lasted for 22–30 s for thieves and 12–16 s for victims.

**Table 3:** Observation time and facial viewing time in stimulus films used in Experiments 1 and 2

	Standard observation time (s)				Enhanced observation time (s)			
Film version	Film 1.1		Film 1.2		Film 1.3		Film 1.4	
Role (actor)	Thief (A)	Victim (B)	Thief (B)	Victim (A)	Thief (A)	Victim (B)	Thief (B)	Victim (A)
Experiment 1								
Frontal face view	12	7	9	9	30	12	22	16
Close-ups	16	7	6	9	32	13	22	11
Overall facial viewing time	20	15	15	19	44	26	34	28
Overall viewing time	36	29	30	34	54	45	51	45
Overall film duration (min:s)	1:15		1:14		2:35		2:35	

	Shorter observation time (s)				Longer observation time (s)			
Film version	Film 2.1		Film 2.2		Film 2.3		Film 2.4	
Role (actor)	Thief (A)	Victim (B)	Thief (B)	Victim (A)	Thief (A)	Victim (B)	Thief (B)	Victim (A)
Experiment 2								
Frontal face view	7	5	6	5	20	10	21	10
Close-ups	5	7	5	5	22	10	22	11
Overall facial viewing time	12	12	12	13	34	19	34	22
Overall viewing time	25	26	24	24	44	38	45	39
Overall film duration (min:s)	1:03		00:59		2:09		2:16	

For Experiment 2, we created two film versions with shorter observation time by decreasing facial exposure, close-ups, and overall viewing time. Additionally, we edited the enhanced film versions to decrease variation across film versions (cf. Table 3). In the shorter film versions, overall observation time was approximately 25 s, with 5–7 s of frontal face views. In the longer film versions, overall observation time was 44–45 s for thieves and 38–39 s for victims and frontal faces views were 20–21 s for thieves and 10 s for victims. In other words, for the shorter vs. longer film versions, the frontal face view ratio was approximately 1:3 for thieves and 1:2 for victims.

#### CIT and lineup photos

We used the same facial pictures for the CIT task and lineups. Pictures showed probes, targets, and irrelevants from the collarbone up, without jewelry, eyeglasses, or hair accessories and with loose hair. The pictures fitted the general description of the probes depicted in the different stimulus events, as determined by presenting independent samples of mock witnesses (*n*s between 23 and 31) who had not viewed the stimulus event with a description of each probe (or probe replacement) together with five fillers (e.g., ‘She is about 20 years old. She has long, brown hair. She has a slim to normal figure’). These mock witnesses then selected the person from the lineup who matched the description best (Doob & Kirshenbaum, 1973). If all fillers are good alternatives to the probe, each lineup member should receive an approximately equal number of selections from the mock witnesses. The effective lineup size gives an indication in how far this is the case. Ideally, the effective lineup size should be close to its nominal size—six in our case. The effective lineup size Tredoux’s E ranged from 3.8 to 4.8 (of a possible 6), thereby marking them as a fair picture selection (Tredoux, 1998, 1999).

We made some adjustments to the stimuli for the present study. Their clothing was edited to be black and the probes wore different clothing in the photograph than in the film. To avoid recognition of one probe by a small mole below the eye, we edited the target and irrelevants pictures corresponding to this probe to include a mole as well.

#### Reaction time‑based concealed information test

We presented the CIT protocol, using Inquisit 5.0.14.0 web player (Experiment 1) and Inquisit 6.3.3 web player (Experiment 2). The software recorded reaction times in milliseconds. All stimuli pictures were 416 × 520 pixels. We used one joint CIT protocol for the thief and the victim, with the images for thief and victim intermixed. Participants received the instruction to press the right shift key as fast as possible in response to a facial stimulus, with the exception of the two targets. For these stimuli, they should press the left shift key rather than the right one. The targets were randomly selected from the filler faces and always included one picture that matched the thief actor and one that matched the victim actor. Participants viewed the targets for 30 s, accompanied by instructions to encode these faces. In one practice block showing each of the stimuli (probes, targets, fillers) once, participants received feedback (*good*, *wrong*, or *too slow*). Participants had 1500 ms to react before the next stimulus was shown following an inter-stimulus interval of 1000 ms (in Experiment 1) or a random inter-stimulus interval between 250 and 750 ms (In Experiment 2). Participants completed a second practice block if they had more than 50% errors or misses in the first practice block. In this case, the targets were shown for five more seconds and participants were reminded of how they should respond. After this second practice block, participants continued to the actual task regardless of performance.

During the actual task, every stimulus appeared 21 times, the sequences being random. In Experiment 1, participants received feedback for wrong or too slow responses. In Experiment 2, participants did not receive feedback. In Experiment 1, the CIT stimuli consisted of 2 * 7 pictures (2 probes, 2 * 5 fillers, 2 targets). In Experiment 2, we constructed a probe-present and a probe-absent CIT version, consisting of 2 * 6 pictures each. In the probe-present condition, these pictures included the two probes (thief and victim), four fillers, and two targets. In the probe-absent condition, the pictures included the probe replacement (i.e., innocent theft suspect/ replacement for the victim), four fillers, and two targets. We considered a randomly selected irrelevant stimulus as the probe. CIT protocols in Experiment 2 were either probe-absent or probe-present for both the thief and the victim. The resulting number of trials was 294 in Experiment 1 and 252 in Experiment 2. The question “Do you recognize her?” appeared above every stimulus and the labels “YES” and “NO” on the left and right sides. The use of the left vs. right shift key was randomized across participants.

##### Follow-up photo display

Participants in the CIT condition were shown a photo recognition display after the CIT ask. The display included all 14 pictures. Participants had to indicate the individuals they (explicitly) recognized from the stimulus film. This allowed us to roughly determine if participants in the CIT conditions had explicit memory of the probes.

#### Lineups

We composed separate thief and victim lineups of six photographs. In the probe-present conditions, photographs included the probe (i.e., guilty suspect) and five irrelevants (Experiments 1 and 2). In the probe-absent conditions, photographs included the probe replacement and five irrelevants (Experiment 2 only). Lineup members were numbered 1–6, with the numbers arranged in two rows of three pictures (i.e., a simultaneous lineup). Participants were informed that “police are trying to identify the thief from the film you just saw. Because you saw the theft, they present you with a lineup. Note that the thief may or may not be present in this lineup. If you are not sure or do not know, you can select the "not present" option.” For the victim lineup, the instructions were as follows: “You will now view a lineup referring to the victim. Note that the victim may or may not be present in this lineup. If you are not sure or don't know, you can select the "not present" option.” Participants also indicated how confident they were about their identification decision on a scale from 0 to 100% after each lineup. We have not analyzed or reported the confidence data. The sequence of the lineups was fixed (thief–victim), but thief and victim actors were counterbalanced. In Experiment 2, probe presence was fully manipulated across the thief and victim, that is, participants might view both lineups as probe-present, one probe-present and one probe-absent, or both as probe-absent.

#### Attention check

Participants answered three attention check questions, namely two multiple-choice questions with six response options (“What was stolen?”; “Where did you last see the thief in the video?”) and a lengthy text that ended with the instruction to ignore the question (i.e., the correct response was to continue without making a selection). We excluded participants if they did not answer at least two of the three questions correctly.

### Procedure

Testing occurred online, using the university’s research participation system SONA (Experiment 1) or the platform Prolific (Prolific.co; Experiment 2). Participants received instructions to use a PC or laptop, but not a phone or tablet, in a quiet space without disruptions. After providing consent, participants were randomly assigned to one of four (Experiment 1) or eight (Experiment 2) conditions. In Experiment 1, participants in the lineup condition were instructed to watch the following film closely and to pay attention to every detail, whereas participants in the CIT condition were asked to pay close attention to the faces of the people involved. This unintended difference in the instruction was corrected in Experiment 2, where all participants received the instruction to pay attention to every detail. After watching the video, participants answered the three attention check items and then received the CIT task and follow-up photo display or the lineups. After the testing sessions that lasted approximately 20–30 min, participants received the debriefing and reimbursement (Experiment 1: course credit; Experiment 2: £1.35/ 2.10 for lineup/CIT condition; based on duration of participation).

### Analyses

#### RT-CIT

In Experiment 1, using JASP 0.14.1, we conducted a mixed measures 2 × 2 ANOVA with observation time (standard vs. enhanced) as between-subjects factor and stimulus types (probe vs. irrelevants) as within-subjects factor.^3^ We included correct reaction times only (i.e., excluding behavioral errors) and those that occurred in the time frame between 150 and 1500 ms (following Sauerland et al., 2019). In Experiment 2, we conducted a mixed measures 2 × 2 × 2 ANOVA with observation time (shorter vs. longer) and probe presence (present vs. absent) as between-subjects factors and reaction times to different stimulus types as within-subjects factor.

#### Lineups

We conducted 2 × 2 chi-square tests to establish the effect of observation time on identification accuracy. We conducted separate tests for the thief and the victim. In Experiment 2, we computed separate tests for probe-present and probe-absent lineups and additionally report Bayes factors.

#### Comparison of CIT vs. lineup performance

To compare performance in CIT vs. lineups, we classified CIT performance as accurate or inaccurate, based on an individual effect size (*d*CIT). Following earlier work (Kleinberg & Verschuere, 2015) we calculated *d*CIT as [(*M* probe RT—*M* irrelevant RT)/*SD* irrelevant RT]. For probe-present conditions (Experiments 1 and 2), we classified participants with *d*CIT scores > 0.20 as correct and participants with *d*CIT scores ≤ 0.20 as incorrect. For the probe-absent condition (Experiment 2), we classified participants with *d*CIT scores > 0.20 as incorrect and participants with *d*CIT scores ≤ 0.20 as correct. Next, we compared performance (correct vs. incorrect) in the CIT and lineups by means of a 2 × 2 chi-square test and reported Bayes factors.

## Results

### CIT

**Fig. 1:**
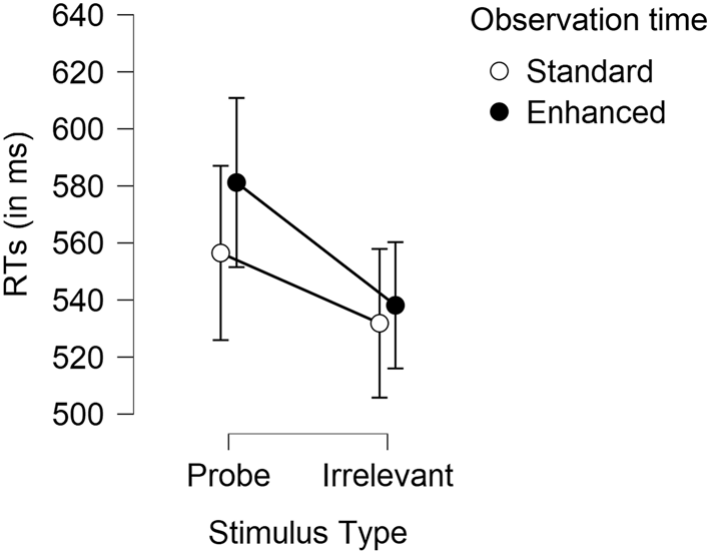
Average mean reaction times to probes and irrelevants (and 95% CI) in the CIT under enhanced vs. standard conditions in Experiment 1

In Experiment 1 (probe-present only), the main effect of stimulus type was significant, *F*(1, 45) = 34.68, *p* < .001, $\eta_{{\text{p}}}^{2}$ = .44, with slower responding to probes (*M* = 569 ms; *SD* = 71) than to irrelevants (*M* = 535 ms; *SD* = 55), *d* = 0.85 [0.51; 1.18], evidencing a CIT effect. Contrary to hypothesis 1 (CIT encoding effect), the interaction between observation time and stimulus type was non-significant, as depicted in Fig. 1, *F*(1, 45) = 2.56, *p* = .117, $\eta_{{\text{p}}}^{2}$ = .05.^4^ The probe-irrelevant difference in RTs was large both for standard observation time, *d* = 0.74 [0.27; ∞] and for enhanced observation time, *d* = 0.97 [0.55; ∞]. The main effect of observation time was non-significant, *F*(1, 45) = 0.77, *p* = .386, $\eta_{{\text{p}}}^{2}$ = .02.

**Fig. 2:**
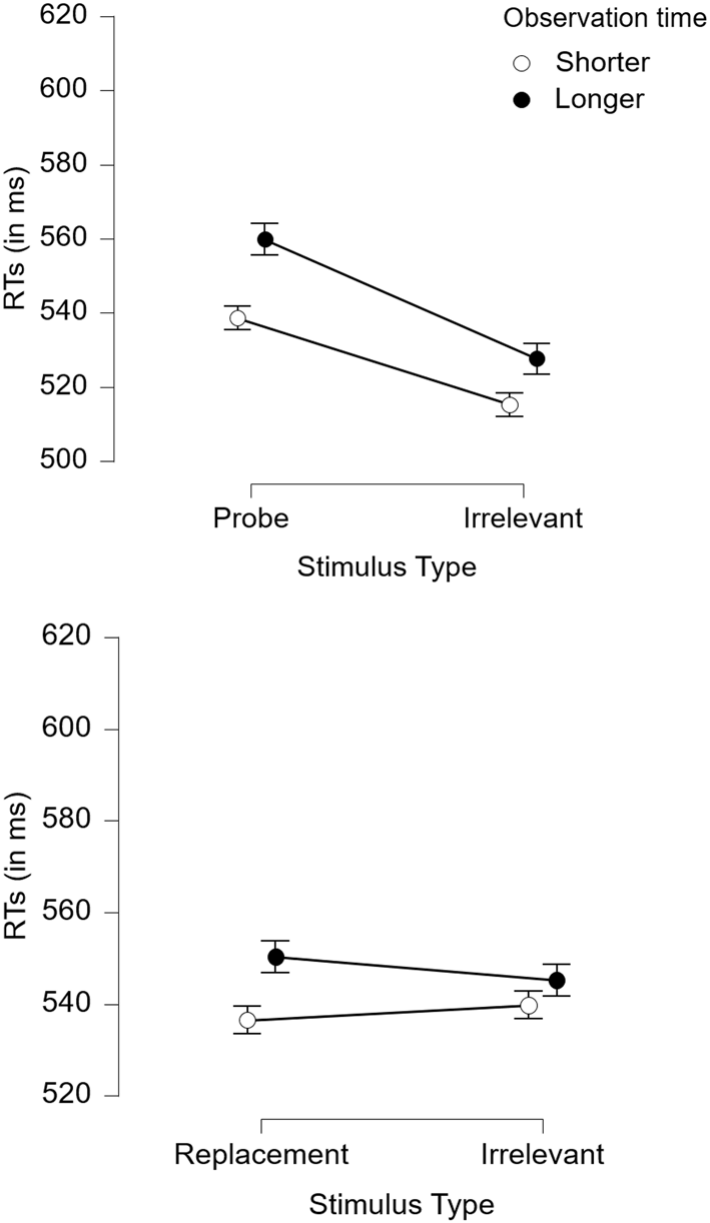
Average mean reaction times to probes or replacements and irrelevants (and 95% CI) in the probe-present (upper panel) vs. probe-absent CIT (lower panel) with shorter vs. longer observation time in Experiment 2

In Experiment 2, the significant main effect of stimulus type, *F*(1, 210) = 34.14, *p* < .001, $\eta_{{\text{p}}}^{2}$ = .14, was qualified by the significant probe presence by stimulus type interaction, *F*(1, 210) = 29.89, *p* < .001, $\eta_{{\text{p}}}^{2}$ = .13. Confirming hypothesis 2, and as illustrated in Fig. 2, a CIT effect emerged in the probe-present condition (*M*_probe_ = 550 ms, *SD*_probe_ = 80 vs. *M*_irrelevant_ = 522 ms, *SD*_irrelevant_ = 64), *t*(100) = 7.42, *p* < .001, *d* = 0.74 [0.52; 0.96], but not the probe-absent condition (*M*_probe_ = 543 ms, *SD*_probe_ = 74 vs. *M*_irrelevant_ = 542 ms, *SD*_irrelevant_ = 71), *t*(112) = 0.19, *p* = .853, *d* = 0.02 [− 0.17; 0.20]. A Bayesian ANOVA with JASP 0.14.1 default settings (i.e., Cauchy priors with *r* scale = 0.5) showed strong support for the probe presence by stimulus type interaction (BF_10_ = 1.6 × 10^6^). The data were 1.6 × 10^6^ times more likely under the model with this two-way interaction than in the model with only the main effects of probe presence and stimulus type.

The predicted three-way interaction was non-significant, *F*(1, 210) < 0.01, *p* = .961, *η*^2^_p_ < .01. The Bayesian ANOVA further provided evidence against the inclusion of the three-way interaction (BF_10_ = 0.15; i.e., the data were 6.7 times less likely under the model that included all main effects, two-way interactions, and the three-way interaction compared to the model that only included the main effects and two-way interactions). Indeed, in the probe-present condition, the CIT effect (difference in reaction times between probes and irrelevants) was moderate to large for both shorter observation time, *d* = 0.76 [0.43; 1.08], and longer observation time, *d* = 0.74 [0.44; 1.05], indicating that the CIT effect was not moderated by observation time (hypothesis 1). This finding is analogous to Experiment 1. All other effects with observation time were non-significant: *F*s(1, 210) ≤ 3.05, *ps* ≥ .083, $\eta_{{\text{p}}}^{2}$ ≤ .01.

#### Lineups

Table 1 shows an overview of identification accuracy rates as a function of observation time. In Experiment 1, identification accuracy did not differ as a function of observation time for the thief, *χ*^2^(1, *N* = 47) = 0.10, *p* = .753, *φ* = .05, or the victim, *χ*^2^(1, *N* = 47) = 0.07, *p* = .798, *φ* = .04. A non-preregistered Bayesian chi-square test suggested that the data were inconclusive (BF_10Thief_ = 0.38; BF_10Victim_ = 0.37).

In Experiment 2, identification accuracy (i.e., correct rejections) was better for longer, compared to shorter observation time in probe-absent lineups for the thief, *χ*^2^(1, *N* = 148) = 10.41, one-sided *p* < .001, *φ* = .27, and the victim, *χ*^2^(1, *N* = 148) = 8.26, one-sided *p* = .002, *φ* = .24. For probe-present lineups, no such effect emerged for the thief, *χ*^2^(1, *N* = 147) = 2.47, one-sided *p* = .058, *φ* = .13, or the victim, *χ*^2^(1, *N* = 147) = 1.35, one-sided *p* = .123, *φ* = .10). These findings partially confirm hypothesis 3. The Bayesian chi-square tests showed that support for the effect of observation time on identification accuracy was strong for probe-absent lineups (BF_10Thief_ = 33.07; BF_10Victim_ = 11.54). The Bayes factor for probe-present lineups suggested that the data were inconclusive (BF_10Thief_ = 0.68; BF_10Victim_ = 0.40).

#### Identification performance in CIT vs. lineups

Table 1 shows the correct classification rates for the CIT and the Bayes factors for the comparison of the two identification procedures as a function of observation time. Overall, the pattern of results was inconsistent, with the Bayes factors indicating there was neither strong support for the null hypothesis (methods are equivalent) nor for the alternative hypothesis (one methods outperforms the other).

### Recognition from follow-up photo display

In both experiments, a binomial test against 1/7 odds (chance level of 0.15) showed that CIT participants identified the thief (*M*_E1_ = 0.74, [0.62; 0.87]; *M*_E2_ = 0.42 [0.35; 0.49]) and the victim (*M*_E1_ = 0.77, [0.64; 0.89]; *M*_E2_ = 0.38, [0.32; 0.45]) above chance level from the photo display, *ps* < .001. Recognition accuracy in this task^5^ did not systematically differ as a function of observation time, with BF_01_ varying between 0.65 and 4.73 (Table S1 in the supplementary materials online shows the correct recognition rates and the Bayes factors for each comparison).

### Deviations from preregistration

We had preregistered to exploratorily analyze Experiment 1 lineup data separately by actor in addition to role. We have not conducted the analyses by actor to restrict the number of analyses and because, in hindsight, these analyses do not make sense from a stimulus sampling perspective (Wells & Windschitl, 1999). We did not preregister the Bayesian chi-square test and Bayesian ANOVA for Experiment 1, and due to an oversight, we had also not preregistered the analyses of the follow-up photo display (Experiments 1 and 2).

## Discussion

In two preregistered experiments, we tested whether a reaction time-based computerized test might serve as a radical alternative to the classic lineup (under favorable encoding conditions). Based on previous experiments (Georgiadou et al., 2019; Sauerland et al., 2019), we expected favorable encoding conditions to improve the capacity of the CIT to diagnose face recognition (CIT encoding effect; hypothesis 1). Extending earlier work, we also included a classic lineup condition as a benchmark of eyewitness performance. While overall capacity to diagnose face recognition by means of the CIT was strong (Cohen’s *d*s between 0.74 and 0.97), observation time did not moderate this effect. In Experiment 2, we further tested the usefulness of an RT-CIT protocol for diagnosing *absence* of face recognition when the perpetrator was absent for the first time (hypothesis 2). As expected, probe presence moderated the CIT effect in that there was a CIT effect for the probe-present (*d* = 0.74 to 0.76), but not for the probe-absent CIT condition (*d* = 0.02). Replicating earlier work (Bornstein et al., 2012), identification performance varied as a function of observation time in Experiment 2 (hypothesis 3), but only for probe-absent (and not probe-present) lineups. Comparisons of identification performance in the RT-CIT vs. lineups were inconclusive.

Essentially, both the CIT and the lineup are memory tests. Generally, better encoding conditions should, through increased memory strength, improve CIT and lineup performance. One possible explanation for our finding that observation time did not (CIT) or only partly (lineup) moderate identification performance could be that shorter observation times were still relatively long, with at least 24 s of overall viewing time, 12 s of facial viewing time, and 5 s of close-ups. Indeed, a meta-analysis on the effect of exposure time on facial recognition accuracy (Bornstein et al., 2012) reported the strongest effects when short observations times were only a few seconds long and the ratio of observation time conditions was 1:4 or higher. While this could be seen as a limitation of this work, we were mindful of creating encoding conditions that still justified an identification procedure. More specifically, we were wary of creating a condition that would render identification performance generally unreliable, as reported, for example at overall viewing times of 12 s (Memon et al., 2003). Rather, we aimed at creating conditions that were in line with the notion that an identification procedure should only take place when the encoding conditions justify a memory test (Wagenaar & Van Der Schrier, 1996). In real live cases, investigators can enquire about the viewing conditions in the prelineup interview (Wells et al., 2020).

For being useful in the field, an identification procedure not only needs to diagnose the presence of face recognition, but also *lack* thereof. In other words, a procedure needs to show that it leads to accurate decisions when the police suspect is guilty, but also when the police suspect is innocent. We therefore, for the first time, included a probe-absent CIT condition in Experiment 2. Reassuringly, the CIT effect in the probe-absent CIT condition was nil (*d* = 0.02; i.e., no statistically significant difference in reaction times to probes vs. irrelevants), compared to strong CIT effects in the probe-present condition in both experiments (Experiment 1: *d* = 0.85; Experiment 2: *d* = 0.74). These findings indicate a good capacity of the CIT to differentiate between guilty and innocent suspects.

Including a lineup condition allowed us to compare correct classification rates in the CIT with identification performance in lineups. Although Experiment 1 may have been underpowered to pick up differences between the two methods, across both experiments, we found no compelling evidence that one method would outperform the other. If future investigations support the notion of equivalence, it could follow that performance of the two procedures is comparable, at least under certain conditions. It is possible, however, that one of the two procedures is superior under certain conditions that have yet to be identified. For example, RT-CIT might be more robust in the face of some impact factors that are known to impair lineup performance. Indeed, in the current two experiments, observation time did not affect RT-CIT performance in a statistically significant way, whereas it did in probe-absent lineups in Experiment 2.

### Limitations and future perspectives

One limitation of this work is that the current two experiments largely used the same stimulus materials as previous experiments—this work was based on (Georgiadou et al., 2019; Sauerland et al., 2019, Experiment 4). The fact that our conclusions are based on a single set of materials (with modifications), warrants replication. On the other hand, the variation in effect sizes across those Experiments points to a susceptibility of the RT-CIT effect for variations in stimuli. For example, compared to Sauerland et al., we edited the clothing in the lineup and CIT photos to be black. Although lineup fairness is similarly vulnerable to bias in the images, the particular clothing worn in lineup pictures is not of the essence, as long as it does not make the suspect stand out (Lindsay et al., 1987; Wetmore et al., 2015). However, for the RT-CIT, variations in clothing color or pattern can provide undesired cues for recognition that affect reaction times, independent of *face* recognition. Similarly, compared to Georgiadou et al. (2019), we edited the target and irrelevant pictures corresponding to one probe to include a mole. Although the effective lineup size for this probe was decent without editing the moles in, it is possible that this discrepancy in the images affected the reaction times in the earlier experiments. Another point worth discussing is that the CIT effects we observed here were well below the average effect size reported in a meta-analysis, namely *d* = 1.04 ([0.93; 1.17], Suchotzki et al., 2017). These large effects are most likely owed to the use of autobiographic, self-relevant stimuli (e.g., participant’s name, address) in memory detection experiments. Additionally, these studies use several groups of stimuli (e.g., weapons, crime locations, names of co-perpetrators), whereas for our purposes, we are limited to faces. Options for enhancing the size of the CIT effect include increasing the number of targets, using familiar targets^6^ (cf. Suchotzki et al., 2018), and increasing the number of probes, for example by adding photographs of different aspects of a person (e.g., body vs. face) or by using other information known about the perpetrator (e.g., clothing, jewelry, or a bag; cf. Pryke et al., 2004; Sauerland & Sporer, 2008). Finally, differences in the lineup vs. CIT instructions (i.e., to pay attention to every detail vs. the faces, respectively) are a limitation of Experiment 1. Clearly, for comparing the usefulness of each method, facial encoding should be identical. Directing special attention to the faces in the CIT but not the lineup might have introduced a bias in favor of the CIT in Experiment 1. Yet, the findings of Experiment 1 and 2 were strikingly similar. Thus, it seems that the differences in the instructions did not have a meaningful impact on the pattern of results.

Thus far, researchers have tested the usefulness of RT-CIT as a means of diagnosing face recognition under pristine conditions in ten experiments (the current work; Georgiadou et al., 2019; Sauerland et al., 2019). One possible next step could be to investigate how the RT-CIT fares—in comparison to lineups—in the presence of bias, for example in the (dis)similarity of facial stimuli or the administration of the procedure. Given the implicit nature of the RT-CIT, the procedure might be less prone to impact factors related to the social situation that unfold during lineup administration, such as experimenter effects and demand characteristics (Wells & Luus, 1990).

## Conclusion

Although the RT-CIT certainly does not constitute a magic wand for identifying perpetrators and discharging innocent suspects, accumulating evidence suggests that it may be diagnostic of facial recognition. This could be relevant for cases with witnesses who do not want to make an explicit identification, as a result of threat or to protect the perpetrator. An interesting avenue for future research concerns possible factors that might facilitate an RT-CIT effect while undermining lineup performance.

## Supplementary Information

Below is the link to the electronic supplementary material.

Supplementary file1 (DOCX 16 KB)

## Footnotes

1 Unlike the terminology in the lineup literature, in the CIT, the term *target* describes the person to whom the participant has to react differently. The term *probe* describes the person seen during the stimulus event.

2 In hindsight, we realized we should have used the expected effect size for the interaction effect between stimulus type and observation time rather than the expected effect of stimulus type. In a meta-analysis, the weighted mean effect size of observation time on identification accuracy was *d* = 0.63 (Bornstein et al., 2012). Assuming a similar effect of observation time on the capacity of the CIT to diagnose face recognition, if anything, our sampling strategy was conservative.

3 Because error rates are typically very low (here: 1% for foils, and 2% for probes) and lead to less reliable results (Kleinberg & Verschuere, 2015), we preregistered to test our hypotheses exclusively on RTs.

4 A non-preregistered Bayesian ANOVA with JASP 0.14.1 default settings (i.e., Cauchy priors with r scale = .5) showed that the model that included the two main effects and the interaction was not more likely than the model with only the two main effects (BF_10_ = 0.83). With the BF being close to 1, the data of Experiment 1 neither spoke for nor against the inclusion of the interaction.

5 The purpose of the follow-up photo display was to check that encoding of the probes during the film took place, and it should not be regarded as an additional lineup. There are important differences between the follow-up photo display and a lineup. First, the follow-up photo display concerns a forced choice task that does not include an option to reject. Furthermore, because the CIT task preceded the follow-up photo display, it should be considered a repeated identification procedure. Recommendations for lineup procedures rightfully advise against repeated identification attempts with the same witness and the same suspect (Wells et al., 2020), because the second identification attempt does not have diagnostic value. This is because the preceding procedure familiarizes participants (witnesses) with the stimuli presented in the photo display and possibly introduces unconscious transference effects (Deffenbacher et al., 2006). As a result, the accuracy rates for follow-up photo displays can be inflated.

6 Note: familiar *targets*, not familiar *probes*.

## Declarations

### Human and animal participants

All procedures performed in studies involving human participants were in accordance with the ethical standards of the institutional and/or national research committee and with the 1964 Helsinki Declaration and its later amendments or comparable ethical standards.
