# Supplementary material for: Diagnosing eyewitness identifications with reaction time-based concealed information test: the effect of observation time
Source: Psychol Res. 2022 Feb 8;87(1):281–93. doi: 10.1007/s00426-022-01643-5 (PMC9873779; doi:10.1007/s00426-022-01643-5)
Supplement: Supplementary file 1 — Supplementary file1 (DOCX 16 KB) [file 426_2022_1643_MOESM1_ESM.docx]

**Table 1**

*Correct Recognition Rate From the Follow-Up Photo Display and Bayes Factors as a Function of Observation Time*

| Experiment 1  (*N* = 47) | Standard  observation time | Enhanced  observation time | Bayes factor ^a^ | |
| --- | --- | --- | --- | --- |
|  |  |  | BF_10_ | BF_01_ |
| Thief | 78.3 | 70.8 |  | 2.79 |
| Victim | 69.6 | 83.3 |  | 1.87 |
| Experiment 2  (*N* = 211) | Shorter  observation time | Longer  observation time | Bayes factor | |
|  |  |  | BF_10_ | BF_01_ |
| Thief | 35.2 | 49.5 | 1.53 |  |
| Victim | 36.1 | 40.8 |  | 4.73 |

**Note***:* Recognition rates from the follow-up photo display are not suited for comparison with correct identification rates in the lineup condition. There are important differences between the follow-up photo display and a lineup, including the lack of an option to reject and that the follow-up photo display concerns a repeated identification procedure (see footnote 5 in the manuscript for more detail).

^a^ The Bayes Factor BF_10_ expresses how much more likely the data are under the hypothesis of a difference in recognition performance as a function of observation time compared to the null hypothesis of no difference as a function of observation time. If the evidence supports the null hypothesis (i.e., BF_10_ < 1), we present BF_01_ for ease of interpretation. BF_01_ expresses how much more likely the data are under the null hypothesis (no difference as a function of observation time) as compared to the alternative hypothesis (a difference as a function of observation time).
